# How to read stone tools: A new mode system for describing variation in the Eastern African lithic record?

**DOI:** 10.1002/evan.21866

**Published:** 2020-10-16

**Authors:** Lucy Timbrell

**Affiliations:** ^1^ Department of Archaeology, Classics and Egyptology University of Liverpool Liverpool UK

THE PREHISTORIC STONE TOOLS OF EASTERN AFRICA: A GUIDE SheaJ. J. 2020 Cambridge: Cambridge University Press. ISBN: 9781108334969. £85.

Classification of stone artifacts is challenging yet essential for understanding hominin behavior in the deep past. *The Prehistoric Stone Tools of Eastern Africa: A Guide* attempts (a) to provide students interested in Eastern African archeology with a simple and straightforward introduction to stone tools; (b) to facilitate intraregional comparative analysis over time and space; and (c) to provide a new framework for investigating evolutionary and historically vital questions about hominins inhabiting the area. It accomplishes these goals exceptionally well, offering both a comprehensive overview of stone tool evidence in Eastern Africa as well as a novel system through which lithics from this region can be examined, the Eastern African Stone Tool (EAST) Typology. The book's author, John Shea, is successful in convincing the reader that the EAST Typology could reform stone tool systematics in Eastern African archeology; his expertise as an experienced stone tool analyst is demonstrated throughout this guide, yet how feasible his proposed overhaul of Eastern African stool tool systematics will prove to be, and how widely his novel framework will be applied, remains to be seen.

Unlike archeological theory and methods, which have seen consistent review, the classification of Eastern African stone tools has not experienced similar development. Multiple systems are presently adopted for categorizing African stone tools of similar age and provenance, such as Africa's 'Three Age System' and Modes 1‐5,^1^, ^2^ though whether these actually reflect patterning in the existing evidence is highly debated, especially as they inevitably undervalue the diversity and complexity of variation in the African archeological record.^3^, ^4^, ^5^ Because of this “lithics system anarchy” (a phrase coined by Shea), many prominent scholars have previously called for a reform in stone tool systematics,^6^, ^7^, ^8^ yet, so far, these have had little, if any, effect on scientific practice. For example, delegates of the 1965 Burg Warstenstein conference proposed that the 'Three Age system'^1^ should be abandoned; however, they failed to produce an effective alternative and this terminology widely remains in use.^3^, ^4^ Despite previous calls for continental‐wide change being largely unsuccessful, Shea's novel typology has good potential to drastically improve stone tool systematics in Eastern African archeology. This is primarily because the author does not demand the rejection of widely used nomenclatures, as have previous reformation attempts, but rather methodologically addresses problematic areas specific to Eastern African archeology, such as redundant named stone tool industries^9^ (also widely known as 'NASTIES'), in order to enhance the accuracy of stone tool categorization and standardize archeological practice in the region. Standardization could ultimately improve the accuracy of comparisons between sites which would greatly enhance our understanding of hominin behavior in the region. One of the main issues with not having a single set of standards through which to describe, classify, measure, or analyze stone tool evidence is that it makes it largely impossible to differentiate between variation deriving from hominin behavior across Eastern African sites and that introduced by semantics; the EAST Typology offers an impartial solution to this issue.

Impressively, this guide offers also one of the most comprehensive syntheses of Eastern African stone tool evidence to date. Despite the ever‐increasing number of archeologists interested in Eastern Africa stone tool archeology and the huge amount of research interest (and funding) dedicated to understanding hominin behavioral evolution in the region, the last major overview of the Eastern African Stone Age record was published in the 1950s.^10^ Shea's handbook therefore fulfills the need for a revised report detailing the current stone tool evidence, as well as an updated evaluation of how this body of evidence should be approached in practice. The EAST Typology borne from this synthesis offers a bespoke approach for the analysis of stone tool data across the expanse of Eastern African prehistory which could be very powerful coupled with transparent quantitative analysis.^11^As noted by the author, it is especially poignant to fully understand behavior in this region due to its pivotal role in understanding long‐term trends in hominin evolution.

## 1 A GUIDE TO THE PREHISTORIC STONE TOOLS OF EASTERN AFRICA

This book has three main sections. The first comprises Chapters 2–3, which provide a basic yet thorough introduction to stone tools and how to 'read' them. This book is targeted at students and professionals somewhat unfamiliar with prehistoric stone tools, therefore the author introduces the essential terms and concepts used to describe, examine, and interpret this type of evidence in these chapters. Shea familiarizes the inexperienced reader with the vocabulary used by archeologists when studying stone tools, as well as introducing them to the current debates in stone tool analyses. The author does well to place the student within these debates, offering advice on best practice and how students can contribute to these long‐standing conversations.

Chapters 4–5 form the second section, describing Eastern Africa and the significance of its stone tool evidence for understanding prehistoric populations. An introduction to Eastern Africa's topography, geology, and environments—all of which influence hominin evolution and behavior in this region—is provided by Chapter 4. Importantly, this chapter also discusses the history of research in Eastern Africa and the current frameworks for the region's prehistory, demonstrating their implications for research today. Chapter 5 centers on the stone tool evidence in Eastern Africa, describing the different artifact types and NASTIES that typify major prehistoric age stages, the use of which—Shea argues^9^, ^12^, ^13^—no longer holds merit based on the updated body of evidence. In Chapter 5, the author compares over 250 archeological collections from the Eastern African archeological record using his previously established Lithic Modes A‐I.^12^, ^13^ The poor correlation between the stone tool evidence and his earlier framework justify the book's centerpiece, the EAST Typology. Shea acknowledges that not all artifacts will fit his EAST Typology (though dismisses this as an argument against its use) and welcomes proposals to recognize new artifact types, providing criteria through which these would be assessed. Such a dynamic mode system is arguably vital in a field like prehistoric archeology whereby single discoveries can lead to considerable overhauls in our understanding of early hominin behavior.

The third and largest section of the book, Chapters 6–9, introduces the EAST Typology. This novel framework describes Eastern African stone tools in terms of nine technological categories (Groups I–IX), within which further subdivisions define more specific artifact types. The hierarchical nature of this typological mode system enables archeologists to recognize consistencies among the many different stone tool typologies currently in use, as well as making it easier and more effectual to compare stone tool evidence across periods and regions. Chapters 6–9 also describe different ways of measuring artifacts, suggesting when different types of analyses should be used, which is particularly useful for inexperienced readers. In conclusion, Chapter 10 considers the wider questions in Eastern African archeology and how studies of stone tools in this region, as well as archeological and academic practice, can be made more relevant and useful to prehistoric research.

Running through this guidebook is a series of short fictional episodes set on an Eastern African archeological excavation in Uwazi Valley. Based loosely on real characters and events, Shea uses humorous dialogue to convey the realities of being an archeologist in this region, with each episode embodying the issues, controversies and topics raised by the author in each chapter. This truly brings the guide to life, making the content of this book more accessible to students with little contextual knowledge and experience through which to understand its content.

## 2 REFORMING EASTERN AFRICAN STONE TOOL SYSTEMATICS

This guide, and the associated EAST Typology, is a welcome addition to the reading list of any student or professional interested in African archeology. The style, content and nature of the book is ideally pitched as an introduction for those with little to no prior knowledge of the Eastern African stone tool record, providing helpful guidance, clear illustrations and detailed descriptions. Its extensive coverage of Eastern African stone tool evidence is outstanding but not overwhelming for beginners due to its simple and straightforward language. The EAST Typology is an easy‐to‐use yet comprehensive mode‐based system that could be easily be adopted by students and professional alike.

The author succeeds in providing a standardized typological system for describing Eastern African stone tools, the absence of which has previously limited research in this region. As the author notes, such a reform in stone tool systematics has the potential to answer some of the most important questions in paleoanthropology, such as how stone working evolved and why it was abandoned in the majority of cases as well as queries about how sites of different ages and geography relate to each other.

However, whether the EAST Typology will become standard practice in Eastern African archeology, as the author optimistically intends, is far from clear. The author himself acknowledges that, historically, archeologists are reluctant to revise established artifact typologies with earlier attempts at reform, including his own Lithic Modes A‐I,^12^, ^13^ proving largely unfruitful. That said, similar recent calls for standardization in other areas of prehistoric archeology^14^, ^15^, ^16^, ^17^, ^18^, ^19^, ^20^, ^21^ suggests that Shea's appeal for abandoning well‐known NASTIES in Eastern African archeology in favor of a system free of presumptions is timely and opportune, despite some reservations about his total rejection of cultural taxonomy.^22^ Therefore, *The Prehistoric Stone Tools of Eastern Africa: A Guide* offers an innovative solution that may be uniquely positioned to revolutionize research in Eastern Africa, should archeologists working in the region be receptive of it.
